# High catalytic activity and pollutants resistivity using Fe-AAPyr cathode catalyst for microbial fuel cell application

**DOI:** 10.1038/srep16596

**Published:** 2015-11-13

**Authors:** Carlo Santoro, Alexey Serov, Claudia W. Narvaez Villarrubia, Sarah Stariha, Sofia Babanova, Kateryna Artyushkova, Andrew J. Schuler, Plamen Atanassov

**Affiliations:** 1 Department of Chemical & Biological Engineering, Center for Micro-Engineered Materials, University of New Mexico, Albuquerque, NM 87131, USA; 2 Department of Civil Engineering, Center for Emerging Energy Technologies, University of New Mexico, Albuquerque, NM 87131, USA

## Abstract

For the first time, a new generation of innovative non-platinum group metal catalysts based on iron and aminoantipyrine as precursor (Fe-AAPyr) has been utilized in a membraneless single-chamber microbial fuel cell (SCMFC) running on wastewater. Fe-AAPyr was used as an oxygen reduction catalyst in a passive gas-diffusion cathode and implemented in SCMFC design. This catalyst demonstrated better performance than platinum (Pt) during screening in “clean” conditions (PBS), and no degradation in performance during the operation in wastewater. The maximum power density generated by the SCMFC with Fe-AAPyr was 167 ± 6 μW cm^−2^ and remained stable over 16 days, while SCMFC with Pt decreased to 113 ± 4 μW cm^−2^ by day 13, achieving similar values of an activated carbon based cathode. The presence of S^2−^ and 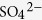 showed insignificant decrease of ORR activity for the Fe-AAPyr. The reported results clearly demonstrate that Fe-AAPyr can be utilized in MFCs under the harsh conditions of wastewater.

Energy and water availability are critical challenges to sustainable development in the 21^st^ century. Treatment of wastewater using available technologies is generally energy-consuming and, consequently, expensive^1^. Microbial fuel cells (MFCs) represent a promising technology for wastewater treatment, while directly generating electrical energy^2^,^3^. Recently, the energy output from MFCs has been successfully applied for powering small electronic devices such as sensors^4^,^5^, pumps^6^, clocks^7^ and mobile phones^8^.

One barrier to long-term application of MFCs in wastewater treatment is the cathode material and design. Existing materials generally suffer from low durability^9^,^10^ (as from poisoning by contaminants), and high costs (as with platinum-based materials)^11^,^12^. The most common and preferred cathode for MFCs and for fuel cells in general is based on an oxygen reduction reaction (ORR), where oxygen is supplied from air. ORR can occur via either 2e^−^ per O_2_ (H_2_O_2_ pathway) or 4e^–^ per O_2_ (H_2_O pathway), with the latter pathway being preferred due to the larger number of electrons transferred and the production of H_2_O as a final product. Cathode overpotential^13^ and catalyst poisoning^10^ are substantial problems that lead to dramatic kinetic losses in ORR in both short and long term operations^14^,^15^. The overpotential is mainly caused by the low catalytic activity of the catalysts in the pH range of 6–8^16^, which is the typical pH range of wastewater.

Despite Pt has been the most utilized catalyst for oxygen reduction reaction at the cathode^17^, Pt is not suitable as a cathode catalyst for MFCs systems^18^. Two different materials have been evaluated as alternative efficient catalysts, one based on carbonaceous materials^19^ and the other one on inexpensive transition metals^20^. In fact, modified carbonaceous materials (e.g. activated carbon and activated carbon nanofibers) possess interesting characteristics of high surface area^20^, high electrical conductivity^21^, high durability^22^, biocompatible capacity for enhanced bacteria attachment and biocathode formation^23^, and low cost^24^ that makes them promising and efficient catalysts for use in MFCs. Development of inorganic catalysts based on inexpensive transition metals (e.g. Co^25^,^26^,^27^, Fe^27^,^28^,^29^,^30^ and Mn^30^,^31^,^32^) categorized as non-platinum group metals (non-PGM) catalysts is another promising alternative. To explore this alternative cathodes with non-PGM catalyst, applied in an air-breathing gas diffusion electrode design and incorporated in membraneless single-chamber MFC (SCMFC) were investigated in this study. A non-PGM catalyst based on low cost iron-aminoantipyrine (Fe-AAPyr) as a precursor for sacrificial support method (SSM) of synthesis was, for the first time, used as SCMFC cathode. Single electrode performances over time were characterized and compared with platinum-based and activated carbon (AC)-based cathodes. The cathodes’ performance was investigated for 16 days, and the benefits of utilizing non-PGM cathode catalyst was demonstrated. Catalyst poisoning tests were conducted using pollutants commonly present in wastewaters (S^2−^ and SO_4_^2–^). Fe-AAPyr performed better than platinum (Pt) during the initial screening in “clean” conditions and showed no degradation in performance during long-term MFCs operation while exposed constantly to pollutants of real wastewater.

## Results and Discussion

Three gas-diffusion cathodes composed of a catalytic layer sprayed onto a teflonized activated carbon, gas-diffusion layer (GDL) were evaluated in both “clean” conditions (PBS) and with real wastewater. The performances of Pt, activated carbon (AC) and Fe-AAPyr as cathode catalysts were compared. The first two of these have been commonly used in MFC cathodes^17^,^18^. Fe-AAPyr is a recently developed catalyst for ORR employed in “inorganic” fuel cells^33^,^34^,^35^,^36^, and has been successfully utilized in a double chamber MFC^37^. The advantage of a double-chamber MFC for wastewater purification is the ability to have different electrolytes in the cathodic and anodic compartments, which reduces cathode contamination from wastewater pollutants^37^. Double-chamber MFC’s main disadvantage is the increased internal resistance due to the presence of a proton-permeable membrane separating the compartments^38^. In SCMFCs, this membrane is not required, but the cathode is directly exposed to wastewater pollutants and may be poisoned more quickly. The latter creates the need of finding a catalyst, which will sustain its activity under the severe conditions of wastewater. A SCMFC was used in the current study and the short and long-term operation of the three types of cathodes at various conditions was monitored.

### Surface Morphology

**Figure 1:**
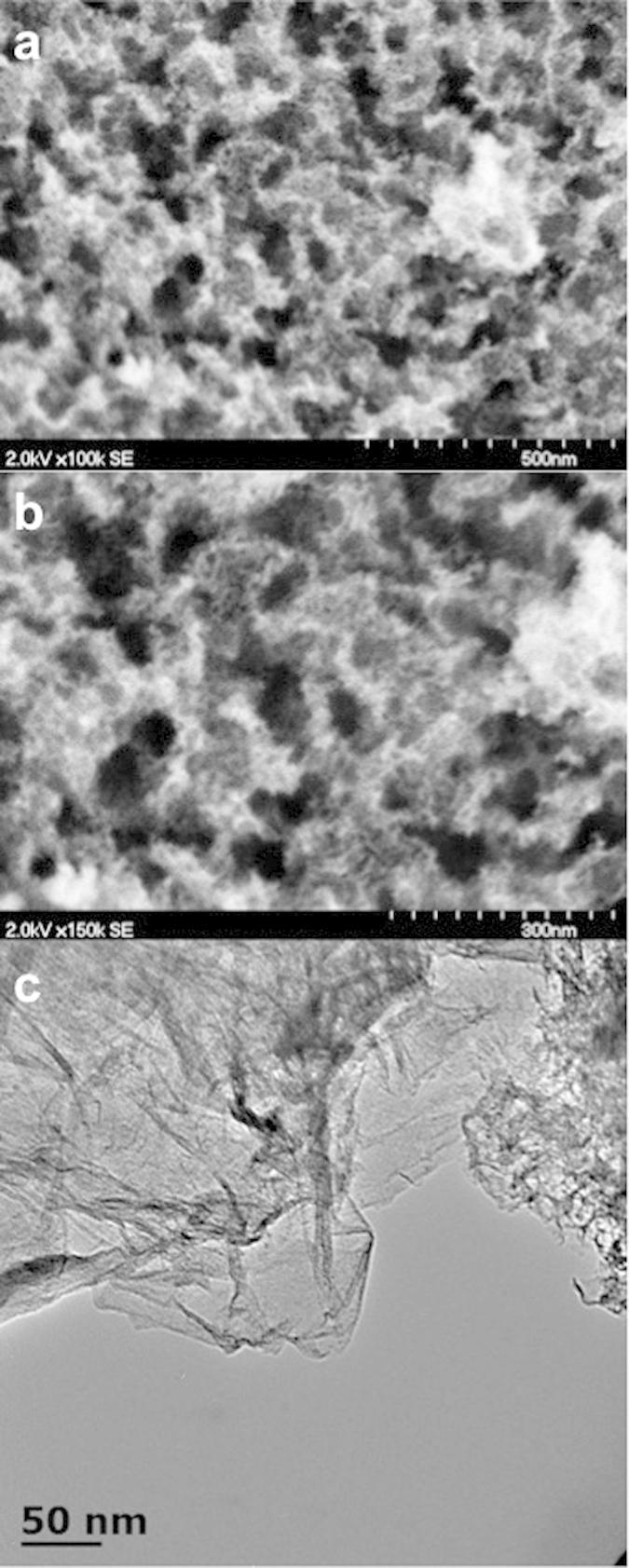
SEM images of Fe-AAPyr prepared by SSM at 100 k magnification. (**a**) and 150 k (**b**). TEM image of Fe-AAPyr prepared by SSM (**c**).

Morphological analysis of Fe-AAPyr catalyst by SEM revealed that the material possesses a highly developed 3D open-frame structure (Fig. 1). Two types of pores can be seen at higher magnification (Fig. 1b): pores with diameter ~60–90 nm were created after removal of the sacrificial support, while smaller pores ~10–15 nm were formed during the decomposition of aminoantipyrine. TEM image of Fe-AAPyr is shown on Fig. 1c. The catalyst has heterogeneous morphology with clear indication of a highly graphitic, high surface area, three-dimensional, graphene-like structure typical for SSM catalysts^33^,^34^,^35^,^36^. This 3D open-frame structure provides better contact of the reacting species with the catalyst active centers and thus enhances current performance.

### Single Electrode Performance in “Clean” Conditions

**Figure 2:**
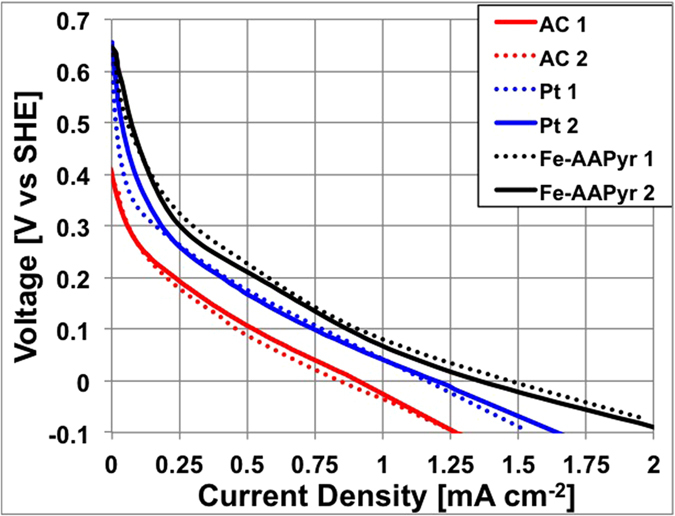
LSVs of the three types of cathodes investigated: Fe-AAPyr (black), Pt (blue) and AC (red) in clean conditions.

Linear sweep voltammetry (LSV) was performed in PBS solution with pH 7.5 (“clean” conditions) as to compare the electrocatalytic activity of the catalysts without the influence of any additional factors. Pt was included as a benchmark, since it is considered to be the most active catalyst for ORR^17^,^18^. Before the test, the cathodes were exposed to the PBS solution for at least 12 hours, until the open circuit potential (OCP) was stabilized, to achieve complete wettability of the catalyst. Initial cathode OCPs for Pt and Fe-AAPyr were similar, 630 ± 18 mV (vs. SHE) and 637 ± 8 mV (vs. SHE) respectively. Much lower values were measured for the AC-based cathode (402 ± 10 mV vs. SHE). It must be noticed that the theoretical potential for ORR in neutral pH (pH = 7.5) is ≈786 mV (vs SHE). This means that the activation overpotentials were ≈155 mV for Fe-AAPyr, ≈149 mV for Pt and ≈384 mV for AC cathode. The Fe-AAPyr cathode demonstrated slightly higher cathodic activity than Pt, and much higher activity than AC, based on LSVs carried out in PBS (Fig. 2). The current densities achieved in this study are representative for ORR in neutral media and as such were much lower in comparison to the current produced by passive air breathing gas diffusion cathodes working in acid or alkaline media^39^. This is due to the low catalysts activity at neutral pH, which has been the least studied in literature.

At last, this result differed from that found in a two-chamber MFC, where Pt outperformed the Fe-AAPyr^37^. This discrepancy might be due to the utilization of the Pt and the Fe-AAPyr in this study in a gas diffusion electrode design in contrast to the submerged in the electrolyte cathode of the previous study, where different parameters are affecting the cathodes performance^37^.

### Voltage and power generation in SCMFCS with activated sludge addition

**Figure 3:**
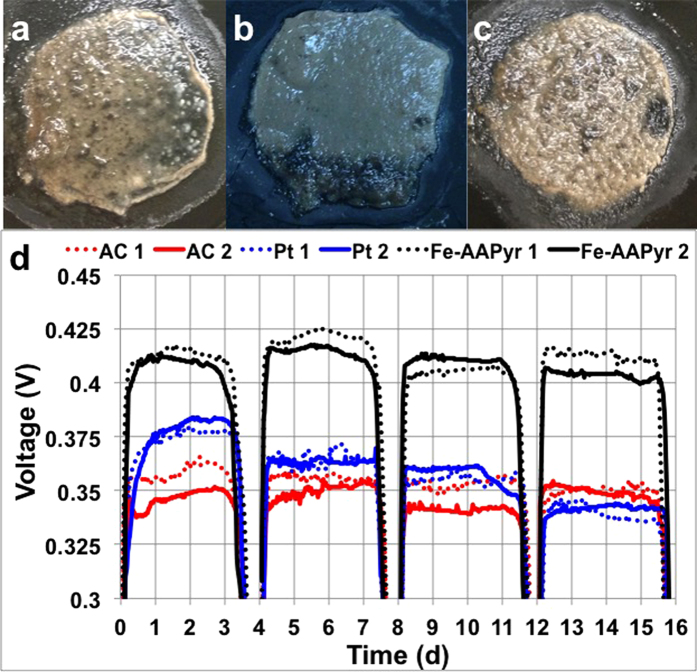
(**a**) Biofilm growth on the cathode with AC (i), Pt (ii) and Fe-AAPyr (iii).(**b**) Voltage trends over a 16-day experiment. The numbers 1 and 2 indicated the replicates tested.

Consistent with the previous results obtained by testing the cathode materials under the relatively “clean” conditions of uninoculated PBS, testing of the 3 cathode materials in SCMFCs with activated sludge feed, including potentially catalyst-poisoning wastewater contaminants, demonstrated superior performance by the FeAAPyr cathode SCMFC, including more stable current production over 16 days. These SCMFCs were operated in sequencing batch mode, with the activated sludge/PBS feed mixture completely replaced each 4 days and under а fixed external resistance of 470 Ω. During the first cycle Fe-AAPyr generated a stable voltage of 412 ± 7 mV (308 ± 6 μA cm^−2^ or 124 ± 3 μW cm^−2^), while the Pt and AC cathodes generated voltages of 375 ± 7 mV (276.6 ± 5.7 μA cm^−2^ or 113.2 ± 3.2 μW cm^−2^) and 350 ± 12 mV (257.2 ± 9.1 μA cm^−2^ or 105.1 ± 4.7 μW cm^−2^), respectively (Fig. 3). The SCMFC with Fe-AAPyr catalyst generated voltage, which was 11% higher than that of the SCMFC with Pt-based cathode and it was 21% greater than the AC cathode system. The SCMFCs with Fe-AAPyr and AC cathodes demonstrated almost unchanged performance over the 4 cycles (16 days), while the SCMFC with the Pt cathodes had a decreasing voltage trend over time, to less than 350 mV by the 4^th^ cycle, which was also lower than the performance of the AC-based SCMFC.

We can speculate, and it seems reasonable, that the decrease in the current during the 4 cycles is due to the complete consumption of COD (1 gL^−1^), which is an important aspect in MFC technology. The higher current being generated form the Fe-AAPyr containing MFC is indication for more efficient COD removal and thus water purification.

**Figure 4:**
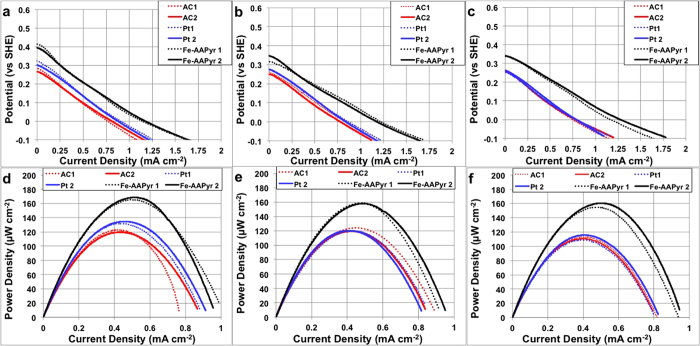
Single Electrode Performance in Operating Conditions after 5 days. (**a**), 9 days (**b**) and 13 days (**c**). Power curves at 5 (**d**), 9 (**e**) and 13 (**f**) days of operation.

The results from intermediate single electrode polarization curves obtained by LSV on the SCMFC’s cathodes during the 16 days study (Fig. 4) were consistent with the overall performances of the systems (Fig. 3). For example, at day 5 (2^nd^ cycle), Fe-AAPyr had a substantially higher electrocatalytic activity in comparison to the Pt and AC cathodes (Fig. 4a,d), while the Pt cathode had slightly higher activity than the AC cathode, which was consistent with the day 5 results shown in Fig. 3. Comparison of the results from Figs 2,4a and indicates that after 5 days of operation in activated sludge, the Pt activity decreased over time, from slightly lower than Fe-AAPyr at day 1 to slightly higher than AC at day 5. At days 9 and 13, the Pt and AC cathodes’ activities were identical. Both, the Fe-AAPyr and AC cathodes had relatively stable electrocatalytic activity during the experiment, showing advantages in long-term durability. These results are consistent with previous work showing that Pt looses activity during long-term operation (1 year) in a microbial fuel cell with PBS alone and without real wastewater or activated sludge^14^. Even faster degradation in platinum performance was observed with the introduction of activated sludge into the electrolyte^10^. The results shown in Figs 3,4 suggest that the Fe-AAPyr cathode may provide the advantages of Pt in terms of high rates of activity, and those of AC in terms of high durability.

Similarly, power density measurements (Fig. 4d–f) were generally consistent with the cell voltage (Fig. 3) and the cathode electrode polarizations (Fig. 4a–c). Anode polarization curves have also been studied (Supporting INFO). It can be noticed that the anode polarization curves started at around −0.3 V (vs SHE) at values close to the theoretical OCP for the anode acetate oxidation reaction indicating negligible activation overpotentials. Moreover, the lower slope of the anode polarization curve compared to the cathode polarization curve underlined that the cathode is the limiting electrode in the studied configuration. It can be also noticed that the anodes performance did not change across the MFCs tested, confirming that the differences were due to the cathode operation. The maximum power density observed for the SCMFC with Fe-AAPyr cathode was 167 ± 6 μW cm^−2^ (day 5), 159 ± 3 μW cm^−2^ (day 9) and 158 ± 8 μW cm^−2^ (day 13). The maximum power observed from SCMFC with the Pt-cathode was 134 ± 4 μW cm^−2^ (day 5), 118 ± 4 μW cm^−2^ (day 9) and 113 ± 4 μW cm^−2^ (day 13), demonstrating a marked decrease from day 5 to day 9. The power densities of the Pt-based cathode SCMFC on days 5 and 9 were similar to those of the AC cathode SCMFC. The power of the AC cathodes MFC remained stable around 117 ± 11 μW cm^−2^ throughout the entire test and is comparable to previously reported values obtained under similar working conditions^20^,^40^. Those results showed that Pt is deactivated in a very short period of time, in fact Pt performed comparably with the carbonaceous substrate (AC) utilized to make the cathodes. Those results underlined the low efficiency of platinum in “dirty” working conditions. Platinum is not only very expensive and, consequently, not appropriate in a technology with low current/power generated, but also it is very sensitive to pollutants present naturally in wastewater which quickly deactivate the catalytic site neglecting the advantages of the platinum as a catalyst^10^.

To the best of our knowledge, only Xia *et al.*^41^ and Zhang *et al.*^42^ have worked with composite cathodes based on activated carbon with addition of a non noble metal catalyst^19^. Xia *et al.*^41^ mixed Fe-EDTA with AC, pyrolyzed the powder at 800 °C in argon gas and then pressed the obtained mixture onto a stainless steel mesh. The maximum power density produced was 158 μW cm^−2^ that was 10% higher than the plain AC cathodes performance. This cathode has been also tested during 17 months with losses quantified in roughly 15% compared to the initial value^14^. Zhang *et al.*^42^ electrodeposited γ-MnO2 on the activated carbon. The modified AC-MnO_2_ air cathode had a maximum power of 155 μW cm^−2^ that was 1.5 times higher than the control cathode based on plain AC. In this current work, the power produced was slightly higher (167 μW cm^−2^) than the previously reported works.

### Biofilm presence on the Cathode

After 16 days, the SCMFCs were dismantled, and the cathodes were inspected. Biofilms were clearly visible on the cathode surfaces facing the waste solutions on all three cathodes types (Fig. 3a). Generally, biofilm formation has been considered a negative factor for the final output, but in this case, the AC and Fe-AAPyr cathodes did not suffer from any decrease in generated power despite the biofilm developed. The relatively stable performance of the AC and Fe-AAPyr cathodes suggests that the biofilms did not significantly reduce the cathode’s performance by increasing the resistance of electron or mass transfer. The decrease in Pt-cathode current may be due to catalyst inactivation by pollutants present in the activated sludge.

### Poisoning Tests

Several common wastewater constituents are known to decrease Pt electrocatalytic activity, such as sulfide and sulfate ion^43^,^44^,^45^. However, little is known about how such compounds affect the Fe-AAPyr activity.

**Figure 5:**
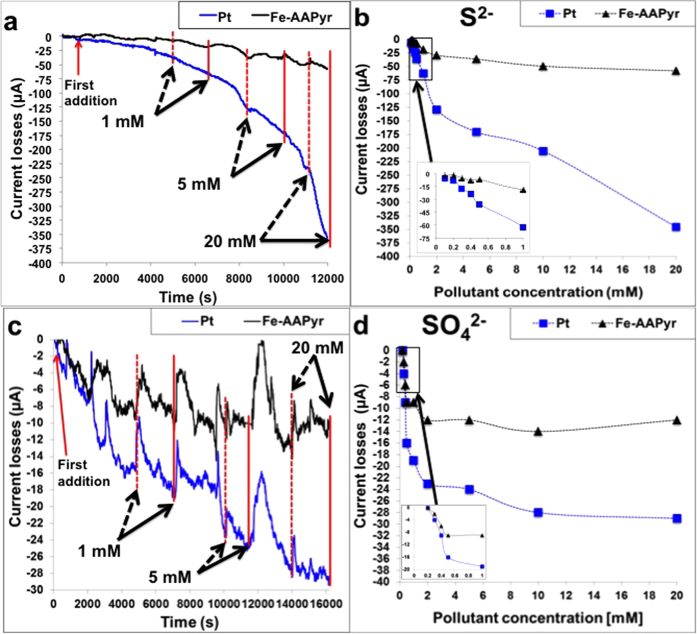
(**a**) Chronoamperometry study with additions of S^2−^(**b**) Current losses in function of the S^2−^ concentration; (**c**) Chronoamperometry study with additions of SO_4_^2−^ (**d**) Current losses in function of the SO_4_^2−^ concentration. Dot arrows represent the pollutant input while continuous arrows represent the value considered for that specific pollutant concentration.

Chronoamperometry measurements of the cathodes at 0 V vs. Ag/AgCl were performed with variable amounts of the sulfide and sulfate ions to monitor the decrease in the ORR current as a result of the pollutants’ inhibition effect. Data were normalized to the initial current in order to underline the current losses over time. Fig. 5a shows the current-time dependence of the tested cathodes as a response to various concentrations of S^2−^. The presence of S^2−^ decreased the activities of both cathodes, with a dramatically higher impact on the Pt-based electrode. Pt cathode lost roughly 36 μA, 170 μA and 345 μA at S^2−^ concentrations of 0.5 mM, 2 mM and 20 mM (Fig. 5). The effect of S^2−^ on Fe-AAPyr cathode performance was much lower, roughly 7 μA, 36 μA and 57 μA at the same S^2−^ concentration of 0.5 mM, 2 mM and 20 mM, respectively. The addition of 20 mM S^2−^ led to a decrease in current that was 6 times lower using Fe-AAPyr compared to Pt (57 μA and 345 μA respectively) showing that Fe-AAPyr catalyst is more tolerant to S^2−^. The effects of 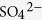 (Fig. 5c,d) were lower in terms of current losses for both of the cathodes tested. At 20 mM 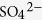 concentration, Pt lost roughly 30 μA while Fe-AAPyr lost only 13 μA. With both chemical species, Fe-AAPyr was more resistant to deactivation than Pt, consistent with the data in Figs 3,4 supporting the practical use of this catalyst in “severe” conditions typical for MFCs treating wastewater.

### Effect of Pollutants on Catalyst Surface Chemistry

X-ray Photoelectron Spectroscopy was used to estimate the changes that occur during exposure of the electrocatalysts to S^2−^ and 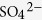 (Table 1). Elemental composition shows that for both of the types of catalysts, there is an increase in overall carbon and loss in F and S, especially for electrolyte containing S^2−^. The changes in the ionomer-catalyst interaction in the cathodes were evaluated from the chemical speciation of sulfur, fluorine and carbon before and after the exposure to the deactivating chemicals.

In beginning-of-life (BOL) Pt and Fe-AAPyr samples, S 2p as two types of chemical environments specific to the ionomer used (Nafion®) at 169.2 and 171.6 eV. After the exposure to pollutants, two new peaks were detected in S 2p spectra, which were identified as sulfur coordinated to carbon (164 eV) and sulfur coordinated to oxygen (166.7 eV) pointing towards deterioration of the ionomer and disruption of the ionomer-catalyst interaction. For Pt-based electrocatalysts, a very small change in sulfur speciation was observed after 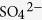 exposure, and this correlates well with the small losses in performance for this type of pollutant in comparison to S^2−^ treatment. Overall chemical changes introduced in S speciation of Fe-AAPyr catalysts were similar to those observed in platinum, while the performance losses for Fe-AAPyr were much smaller than for Pt. Thus the presence of S^2−^ in the electrolyte causes large deterioration of the ionomer composition in both Pt and Fe-AAPyr catalyst layers, but Fe-AAPyr catalysts still retained their activity in a higher degree than Pt electrocatalysts did.

The type of fluorine that is present in the ionomer can add more insight into pollutant action, as fluorine itself is not part of the pollutant as sulfur is, and it is not being introduced during exposure to the solutions. Both Pt and Fe-AAPyr BOL catalysts had similar fluorine composition with C-F (688.7 eV) and CF_2_ (690.3 eV) as expected for Nafion. During testing, oxidation of CF_x_ chains of ionomer is occurring, resulting in two new peaks identified as CxFyOz at higher binding energy of 692–694 eV. Larger chemical changes in the fluorine environment of the ionomer are observed for Pt-based cathode than for Fe-AAPyr, which is correlated with larger losses in the performance for Pt-based electrocatalyst.

In C 1s speciation, oxidation changes of species that are present in ionomer are evident. The largest change in carbon environment is the decrease in the amount of CFx species that are present in the ionomer. This is accompanied by increase of graphitic carbon and the formation of new peaks at higher binding energy of 293–295 eV due to the oxidation of CFx species. These changes in carbon environment are the largest for the Pt-based cathode. Smallest performance losses observed in sulfate are correlated with smallest oxidative changes in the carbon environment.

To conclude observations from surface chemistry, the largest changes were observed for the Pt-based cathodes manifested in carbon oxidation and ionomer degradation contributing to the highest loss of activity.

## Summary

A non-platinum group metal catalyst based on Fe-AAPyr was evaluated for use in a SCMFC gas-diffusion cathode in pollutants-free and wastewater environments. The Fe-AAPyr cathode was used for the oxygen reduction passively supplied from the air to generate electricity. The electrochemical activities of Fe-AAPyr-, Pt-, and AC-based cathode were compared in “clean” (PBS) and “polluted” (activated sludge) environmental conditions. The Fe-AAPyr catalyst demonstrated better performance than Pt and AC in both conditions tested. The Fe-AAPyr catalyst showed much less performance degradation over 16 days than did Pt when in contact with wastewater pollutants. The SCMFC with the Fe-AAPyr-based cathode generated a maximum power of 167 ± 6 μW cm^−2^ which remained stable over 16 days. Contrarily, the performance of Pt-based cathode decreased from 134 ± 40μW cm^−2^ (day 5) to 113 ± 4 μW cm^−2^ at day 13, which was comparable to the performance of AC-based cathode (117 ± 7 μW cm^−2^). Catalyst poisoning tests demonstrated that activity decreased only slightly after immersion in S^2−^ and 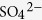. These results suggest that Fe-AAPyr is an excellent catalyst for ORR and for application MFCs for wastewater purification and energy generation.

## Methods

### Cathode materials

Three different cathode catalysts were investigated and compared: i) a platinum-based catalyst, ii) a non-PGM-based catalyst with aminoantipyrine as a precursor (Fe-AAPyr), and iii) AC based catalyst. All three materials had the same support composed of a gas diffusional layer (GDL) built on a carbon cloth as an electron acceptor and a mixture of AC/PTFE pressed on the top of it. In the case of materials i) and ii), an additional catalytic layer was applied while in case of iii), the AC was working as a catalyst.

The non-PGM catalyst included iron and aminoantipyrine as precursors (Fe-AAPyr). Initially, a dispersion of silica (Cab-O-Sil™ LM150, ~200 m^2^ g^−1^, giving a metal loading on silica of 25 wt%) in acetone was obtained by using a low-energy ultrasonic bath. A solution of 4-aminoantipyrine (Sigma-Aldrich) in acetone was separately dispersed in acetone and then added to the silica colloidal solution and ultrasonicated for an additional 40 minutes. Iron (III) nitrate (Fe(NO_3_)_3_*9H_2_O, Sigma-Aldrich) was firstly diluted in distilled water and then added in the SiO_2_-AAPyr solution and ultrasonicated for roughly 8 hours. The gel formed at the end, containing SiO_2_-Fe-AAPyr, was dried for 12 hours at controlled temperature (85 °C) and then grounded to a fine powder using a mortar and pestle. The sample was heated with a temperature ramp rate of 25 °C per minute from room temperature to 950 °C, followed by pyrolysis for 30 minutes. The heat treatment was done in Ultra High Purity (UHP) nitrogen with a flow rate of 100 ml min^−1^. Finally, the silica sacrificial support was removed using hydrofluoric acid (20 wt.%) and the catalyst was washed in distilled water and dried for 12 hours at 85 °C.

### GDL preparation

The cathode support was prepared using a gas diffusion electrode design as previously described^20^. Commercial PTFE-treated carbon cloth (30%wt PTFE, Fuel Cell Earth) was used as an electron collector^20^. On top of it, a mixture of commercial AC (BET area of 802 m^2^ g^−1^, Calgon, Pittsburgh, PA) and PTFE dispersion (60% dispersion in water, Sigma Aldrich) was mixed using a blender^20^. The AC/PTFE ratio was 80/20 wt.%^20^. The AC/PTFE mixture was weighed, placed on the carbon cloth (loading of 60 ± 2 mg cm^−2^) and then pressed at 1400 psi for 5 minutes. After being pressed, the electrode was heated at 200 °C for 1 hour.

### Catalytic payer preparation

Inks of Fe-AAPyr and Pt were prepared by mixing the catalyst (120 mg) with Nafion® (45 wt%) and isopropanol (IPA). The IPA was added in order to reach a solution volume of roughly 7 mL. The inks were then ultrasonicated for 1 hour.

The cathode support, based on carbon cloth with pressed AC/PTFE mixture, was taped on a hot plate with a controlled temperature of 60 °C and the ink was applied on the surface using an air brush spray gun. The temperature of 60 °C allowed fast evaporation of IPA. The change in electrodes weight between initial weight and weight after ink spray, allowed a determination of the catalyst loading. The loading was calculated dividing the change in weight due to the catalyst by the sprayed surface area. The catalyst loadings onto the cathode surfaces were 2.1 ± 0.3 mg cm^−2^ (Fe-AAPyr) and 0.2 ± 0.15 mg cm^−2^ (Pt).

### Materials Surface Analysis

Scanning Electron Microscopy (SEM) and Transmission Electron Microscopy (TEM) were used to determine the morphology of the synthesized catalysts. SEM and TEM images gave important information on the bulk morphology and the individual particle distribution of the analyzed catalyst. SEM images were acquired using an S-3700, Hitachi, Japan. Additionally, TEM images were acquired using a JEOL 2010 microscope with an accelerating voltage of 200 kV and a current of 190 μA.

Surface chemistry of the catalyst before and after the poisoning tests were carried out using X-ray photoelectron spectrometer (XPS) with a Kratos Axis Ultra DLD XPS using a monochromatic Al Kα source operating at 300 W. Survey and high-resolution F 1s, C 1s, O 1s, N 1s and Fe 2p spectra were acquired at pass energies of 80 and 20 eV, respectively. Three areas per sample were analyzed. No charge compensation was necessary. Data analysis and quantification were performed using the CASAXPS software. A linear background was used for F 1s, C 1s, N 1s, and O 1s, while Sherley background was used for Fe 2p spectra. Quantification utilized sensitivity factors that were provided by the manufacturer. A 70% Gaussian/30% Lorentzian (GL(30)) line shape was used for the curve-fits.

### SCMFC Configuration and operating conditions

A membraneless glassy SCMFC with a volume of 125 ml was used^46^, where the anode and the cathode were exposed to the same electrolyte^43^. The cathodes (geometric area of 2.9 cm^2^) were screwed on a lateral hole using a clamp. With the gas-diffusion cathode described above the carbon cloth faced the air, while the catalyst faced the solution^20^. The anode, composed of a carbon brush (6 × 4 cm^2^ projected surface area), was completely immersed in the solution. The anodes were pre-colonized by mixed cultures bacteria taken from previous experiments^37^. The operating solution was a mixture of phosphate buffer saline solution (PBS, 50 mM and 25 mM KCl) and activated sludge (pH = 7.5 ± 0.1) from Albuquerque Southeast Water Reclamation Facility (New Mexico, USA) with a 1:1 volume ratio^37^. Sodium acetate in concentration of 1 gL^−1^ was used as a fuel source for bacteria at the beginning of each test cycle. The operating temperature was 21 ± 1 °C. The experiments were carried out in Albuquerque, New Mexico which is located at approximately 1600 meters above sea level. At this altitude, the atmospheric pressure is roughly 20% lower than at sea level and consequently the oxygen concentration is lower than at sea level. Lower oxygen concentration can negatively affect the performance of the cathode.

### Electrochemical measurements and analysis

The SCMFCs were operated with a constant load and the anode and the cathode were connected to an external resistance of 470 Ω. The voltage was recorded every 25 minutes (Personal DAQ/56)^40^. Single electrode potentiodynamic polarizations curves of the anode and the cathode separately were measured in a three-electrode configuration with a Pt mesh as a counter electrode (specific area comparable to the electrodes investigated), Ag/AgCl (3M KCl) as a reference electrode and the cathode or the anode as a working electrode, respectively^47^. The polarization curves were performed from OCP to −0.1 V for the cathode and for the anode (from OCP to −0.2 V) with a scan rate of 0.2 mVs^−1^ 47. Before the polarization curves, the SCMFC was disconnected until a steady-state OCP was reached (±3 mV).

The overall MFCs polarization curves were recorded using a potentiostat (Gamry P600) with a scan rate of 0.2 mVs^−1^ 40. In this case, counter and reference channels were short-circuited, and both were connected to the cathode, while the working electrode was connected to the anode. The current-voltage curves were then used to obtain the current-power curves using the Ohm law (P = I*V). The current and power were represented in the form of density referred to the cathode geometrical area (2.9 cm^2^).

### Poisoning tests

Chronoamperometry analyses of the three cathodes were performed at constant voltage of 0 V (vs. Ag/AgCl) using the three-electrode configuration previously described. During the electrode polarization aliquots of the pollutants (S^2−^ and 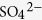) was introduced in the electrolyte measuring the current response. The addition of pollutants varied in the range 0.1 mM and 20 mM. The poisoning effect was calculated as the difference between initial current and current generated after the addition of the pollutant to a given concentration, with the current measured between 15–20 minutes after each addition of each pollutant dose. The current losses were also calculated as function of the pollutants dose.

## Additional Information

**How to cite this article**: Santoro, C. *et al.* High catalytic activity and pollutants resistivity using Fe-AAPyr cathode catalyst for microbial fuel cell application. *Sci. Rep.* **5**, 16596; doi: 10.1038/srep16596 (2015).

## Supplementary Material

Supplementary Information

## Figures and Tables

**Table 1:** Effect of Pollutants on Catalyst Surface Chemistry.

Sample	C %	O %	>F %	S %	Pt %	C-C/ C=C	CxOy	CFx	CxFyOz
Pt BOL	41.7	5.5	52.0	0.67	0.08	28.9	5.4	65.6	
Pt S^2−^	51.1	3.4	45.5	0.19	0.00	39.8	11.4	19.8	28.9
Pt SO_4_^2−^	50.7	5.9	43.2	0.46	0.07	40.4	10.2	37.2	12.2
FeAAPyr BOL	38.4	5.5	54.9	0.59	0.71	13.0	8.4	78.6	
FeAAPyr S^2−^	46.2	2.8	50.9	0.16	0.05	41.1	8.6	34.4	16.0
FeAAPyr SO_4_^2−^	44.3	4.8	50.1	0.47	0.55	62.6	11.1	24.0	2.3
**Sample**	***164.3***	***166.7***	***169.2***	***171.6***		***688.7***	***690.3***	***692.2***	***693.8***
	**S-C**	**S-O**	SO_3_	**CF_3_-S**		**C-F**	CF_2_	**CxOyFz**	
Pt BOL			72.7	27.3		88.4	11.6		
Pt S^2−^	59.3	16.9	19.1	4.7		8.7	15.1	26.1	50.1
Pt SO_4_^2−^	3.6	1.6	61.5	33.4		23.7	37.5	29.7	9.2
FeAAPyr BOL			62.4	37.6		73.7	26.3		
FeAAPyr S^2−^	52.4	14.5	21.3	11.8		13.5	21.6	31.8	33.1
FeAAPyr SO_4_^2−^	39.7	4.4	44.9	11.0		45.1	18.3	22.6	14.0
