# Supplementary material for: High catalytic activity and pollutants resistivity using Fe-AAPyr cathode catalyst for microbial fuel cell application
Source: Sci Rep. 2015 Nov 13;5:16596. doi: 10.1038/srep16596 (PMC4643260; doi:10.1038/srep16596)
Supplement: Supplementary Information [file srep16596-s1.doc]

**Supporting Information**

**High catalytic activity and pollutants resistivity using Fe-AAPyr cathode catalyst for microbial fuel cell application**

Carlo Santoro^1^, Alexey Serov^1^, Claudia W. Narvaez Villarrubia^1^, Sarah Stariha^1^, Sofia Babanova^1^, Kateryna Artyushkova^1^, Andrew J. Schuler^2^, Plamen Atanassov^1^*

*^1^ Department of Chemical & Biological Engineering, Center for Micro-Engineered Materials, University of New Mexico, Albuquerque, NM 87131, USA*

*^2^ Department of Civil Engineering, Center for Emerging Energy Technologies, University of New Mexico, Albuquerque, NM 87131, USA*

**Supporting Figure 1. Anode polarization curves in the range investigated at day 5 (a) and day 13 (b).**
